# Supplementary material for: Long-read sequencing of an advanced cancer cohort resolves rearrangements, unravels haplotypes, and reveals methylation landscapes
Source: Cell Genom. 2024 Oct 14;4(11):100674. doi: 10.1016/j.xgen.2024.100674 (PMC11605692; doi:10.1016/j.xgen.2024.100674)
Supplement: Document S1. Figures S1‒S14 and Tables S2–S6 [file mmc1.pdf]

Cell Genomics, Volume *4*

Supplemental information

Long-read sequencing of an advanced cancer cohort resolves rearrangements, unravels haplotypes, and reveals methylation landscapes

Kieran O'Neill, Erin Pleasance, Jeremy Fan, Vahid Akbari, Glenn Chang, Katherine Dixon, Veronika Csizmok, Signe MacLennan, Vanessa Porter, Andrew Galbraith, Cameron J. Grisdale, Luka Culibrk, John H. Dupuis, Richard Corbett, James Hopkins, Reanne Bowlby, Pawan Pandoh, Duane E. Smailus, Dean Cheng, Tina Wong, Connor Frey, Yaoqing Shen, Eleanor Lewis, Luis F. Paulin, Fritz J. Sedlazeck, Jessica M.T. Nelson, Eric Chuah, Karen L. Mungall, Richard A. Moore, Robin Coope, Andrew J. Mungall, Melissa K. McConechy, Laura M. Williamson, Kasmintan A. Schrader, Stephen Yip, Marco A. Marra, Janessa Laskin, and Steven J.M. Jones

Supplemental Data Tables and Figures

Long-read sequencing of an advanced cancer cohort resolves rearrangements, unravels haplotypes, and reveals methylation landscapes

Kieran O'Neill[1]*, Erin Pleasance[1]*, Jeremy Fan[1]*, Vahid Akbari[1,5]*, Glenn Chang[1]*, Katherine Dixon[1]*, Veronika Csizmok[1]*, Signe MacLennan[1,5,8]*, Vanessa Porter[1,5,8], Andrew Galbraith[1], Cameron J. Grisdale[1], Luka Culibrk[1], John H. Dupuis[1], Richard Corbett[1], James Hopkins[1], Reanne Bowlby[1], Pawan Pandoh[1], Duane E. Smailus[1], Dean Cheng[1], Tina Wong[1], Connor Frey[2], Yaoqing Shen[1], Eleanor Lewis[1], Luis F. Paulin[3], Fritz J. Sedlazeck[3], Jessica M.T. Nelson[1], Eric Chuah[1], Karen L. Mungall[1], Richard A. Moore[1], Robin Coope[1], Andrew J. Mungall[1], Melissa K. McConechy[1], Laura M. Williamson[1], Kasmintan A. Schrader[4,5], Stephen Yip[6], Marco A. Marra[1,5,8], Janessa Laskin[7], Steven J.M. Jones[1,5+]

1 Canada's Michael Smith Genome Sciences Centre at BC Cancer, Vancouver, Canada
2 Department of Medicine, University of British Columbia, Vancouver
3 Human Genome Sequencing Center, Baylor College of Medicine, Houston, TX, USA
4 Hereditary Cancer Program, BC Cancer, Vancouver BC, Canada
5 Department of Medical Genetics, University of British Columbia, Vancouver BC, Canada
6 Department of Pathology and Laboratory Medicine, University of British Columbia, Vancouver BC, Canada
7 Department of Medical Oncology, BC Cancer, Vancouver, Canada
8 Michael Smith Laboratories, University of British Columbia, Vancouver, BC, Canada
*Contributed equally
+Corresponding author

Event Type	SAVANA	nanomonSV	SAVANA and nanomonSV	HQ Illumina	LQ Illumina	HQ and LQ Illumina
Insertion	2(1-4)	58(39-79)	58(39-83)	3(2-7)	93(51-118)	99(52-128)
Deletion	48(25-77)	111(66-175)	119(71-183)	135(98-211)	710(565-758)	862(661-997)
Inversion	43(20-69)	45(25-79)	57(29-89)	48(29-82)	161(142-182)	212(186-269)
Translocation	22(8-32)	45(25-474)	33(20-47)	27(14-46)	190(170-219)	227(197-259)
Duplication	41(23-84)	35(15-80)	49(25-108)	35(15-78)	258(231-287)	297(253-374)

Table S2. Summary of event counts for somatic SVs, related to Figure 2. Values are presented as median (interquartile range, 25th–75th percentile) per sample. LQ = Low Quality. HQ = High Quality.

POG ID	Known Event, (exon, exon)	Event Type	Nanopore Caller Support	Nanopore Coverage (Tumour, Normal)	Illumina Coverage (Tumour, Normal)
POG792	FUS::DDIT3 (e5:e2)	Translocation	cuteSV, Sniffles	36, N/A	85, 41
POG239	MYB::NFIB (e13:e11)	Translocation	cuteSV, Sniffles	34, N/A	89, 45
POG806	EWSR1::PATZ1 (e8:e1)	Inversion	cuteSV, Sniffles	22, N/A	81, 43
POG615	PAX3::FOXO1 (e7:e2)	Translocation	cuteSV, Sniffles	25, N/A	110, 51
POG633	ATG7::BRAF (e18:e9)	Translocation	cuteSV, Sniffles	26, N/A	97, 55
POG662	SS18::SSX1 (e18:e9)	Deletion	cuteSV, Sniffles	15, N/A	76, 37
POG846	NF1::NF1 (e1:e58)	Deletion	cuteSV, Sniffles, nanomonSV, SAVANA	34, 15	74, 33
POG877	EML4::ALK (e13:e20)	Inversion	cuteSV, Sniffles	27, N/A	83, 42

Table S3. Clinically-relevant fusion events and their causal SVs, as called in Illumina data, related to Figure 2. All 8 were recapitulated in the lower-coverage ONT data.

POG_ID	Event coordinate	Gene(s) affected	Event type	Caller support	Nanopore coverage (Tumour, Normal)	Illumina coverage (Tumour, Normal)	Additional comments
POG1002	chr3:128627052-128657344	*RPN1*	Inversion	SAVANA	38, 16	100, 39	Breakpoints in low complexity region
POG782	chr3:128627052-128657344	*RPN1*	Inversion	SAVANA	38, 29	82, 41	Breakpoints in low complexity region
POG137	chr17:39761616-39795054	*IKZF3*	Deletion	SAVANA	32, 27	90, 43	Lack of breakpoint evidence
POG415	chr2:141175402-141277302	*LRP1B*	Deletion	nanomonSV	29, 12	94, 42	Breakpoints in low complexity region
POG117	chr16:18846588-18861404	*SMG1*	Inversion	nanomonSV	38, 28	100, 44	Manually interpreted as a complex event
POG279	chr22:19361162-19366286	*HIRA*	Inversion	SAVANA, nanomonSV	26, 18	89, 41	Manually interpreted as a complex event

Table S4a. Structural variant events called only in ONT (not in Illumina), including notes from manual review, related to Figure 2. Only those overlapping cancer genes were reviewed and included.

**POG ID**	**Event coordinate**	**Gene(s) Affected**	**Event Type**	**Caller support**	**Nanopore Coverage (Tumour, Normal)**	**Illumina Coverage (Tumour, Normal)**	**Additional comments**
POG684	chr22:41164024- 41165548	*EP300*	Deletion	Delly, Manta	21, 23	85, 42	Low coverage
POG303	chr16:72803442-chr4:94786616	*ZFHX3: BMPR1B*	Translocation	Delly, Manta	37, 20	90, 46	Low coverage
POG117	chr3:59901866-59938284	*FHIT*	Deletion	Delly, Manta	38, 28	100, 44	No Nanopore read support
POG117 *	chr2:140878162-chr20:15528198	*LRP1B: MACRO D2*	Translocation	Delly, Manta	38, 28	100, 44	Low coverage

*This event was called two times in the same biopsy sequenced twice, at different times.

Table S4b. Structural variant events called only in Illumina (not in ONT), including notes from manual review, related to Figure 2. Only those overlapping cancer genes were reviewed and included. In 3/4 cases, this was due to low coverage of the ONT sequencing.

POG_ID	Gene Involved	Chr	Position start	Insert sequence size (bp)	Callers	Classification	Results of manual review
POG049	*EGFR*	7	55167011	91	nanomonSV	False positive	Breakpoints are situated in low mapability regions. Presence of multiple insertion signals from 100-500 bp. Event read support in the matched normal.
POG100	*IRF4*	6	396649	109	nanomonSV	False positive	Breakpoints are situated in low mapability regions. Presence of multiple signals from 100-200 bp. Event read support in the matched normal.
POG1022	*RPTOR*	17	80876485	193	nanomonSV	False positive	No read support signal based on visual manual review. Likely an inferred event from assembly. Event read support in the matched normal.
POG111	*KDM2B*	12	121524684	342	nanomonSV	True positive	Improperly classified as an inversion by Illumina read support
POG147	*PTPRD*	9	9985459	223	nanomonSV	True positive	No proximal Illumina calls to breakpoints.
POG295	*TEK*	9	27207649	245	nanomonSV	True positive	No proximal Illumina calls to breakpoints.
POG360	*HDAC4*	2	239069542	206	nanomonSV	False positive	Deletion signal picked up nearby insertion
POG410	*HLF*	17	55282951	59	nanomonSV	False positive	No read support signal based on visual manual review. Likely an inferred event from assembly.
POG530	*TMPRSS2*	21	41474575	539	nanomonSV	False positive	Breakpoints are situated in low mapability regions. Presence of multiple insertion signals from 100-200 bp. Event read support in the matched normal.
POG566	*ARHGAP26*	5	142957710	270	nanomonSV	True positive	No proximal Illumina calls to breakpoints.
POG649	*MAP3K13*	3	185454242	238	nanomonSV	False positive	Breakpoints are situated in low mapability regions. Presence of multiple insertion signals from 100-500 bp. Event read support in the matched normal.
POG649	*NTRK3*	15	88039266	76	nanomonSV	True positive	Missed by Illumina callers.
POG704	*LRP1B*	2	141689497	141	nanomonSV	False positive	No read support signal based on visual manual review. Likely an inferred event from assembly.
POG804	*IGF1R*	15	98908279	319	nanomonSV	True positive	No proximal Illumina calls to breakpoints.

Table S5. Insertion events involving an OncoKB gene unique to Nanopore with manual review comments, related to Figure 2.

POG_ID	gene	chr	pos	ref	alt	Mutant allele	ASE	ASE major allele
POG044	*TERT*	chr5	1295135	G	A	HP2		
POG084	*PLEKHS1*	chr10	113751834	C	T	HP2		
POG109	*PLEKHS1*	chr10	113751834	C	T	HP1	BAE	
POG130	*AP2A1*	chr19	49766782	T	G	HP1		
POG137	*ADGRG6*	chr6	142385069	G	A	Unphased	ASE	HP1
POG153	*ADGRG6*	chr6	142385069	G	A	HP2		
POG170	*ADGRG6*	chr6	142385069	G	A	Unphased	ASE	HP2
POG211	*TERT*	chr5	1295113	G	A	HP1	ASE	HP1
POG217	*ADGRG6*	chr6	142385069	G	A	HP1		
POG415	*TERT*	chr5	1295113	G	A	HP1	ASE	HP1
POG446	*PLEKHS1*	chr10	113751834	C	T	HP1	BAE	
POG497	*TERT*	chr5	1295113	G	A	HP1		
POG574	*TERT*	chr5	1295113	G	A	HP2		
POG574	*AP2A1*	chr19	49766783	C	T	HP2		
POG580	*TERT*	chr5	1295113	G	A	Unphased	ASE	HP1
POG581	*AP2A1*	chr19	49766798	C	T	HP2	BAE	
POG581	*TERT*	chr5	1295113	G	A	HP1	ASE	HP1
POG637	*AP2A1*	chr19	49766781	C	T	HP1		
POG637	*TERT*	chr5	1295113	G	A	HP1		
POG673	*TERT*	chr5	1295135	G	A	HP2	ASE	HP2
POG680	*TERT*	chr5	1295135	G	A	HP1		
POG680	*AP2A1*	chr19	49766726, 49766727, 49766743	G	A	HP2	ASE	HP2
POG693	*TERT*	chr5	1295113	G	A	HP1		
POG777	*TERT*	chr5	1295113	G	A	HP1	ASE	HP1
POG830	*PLEKHS1*	chr10	113751834	C	T	Unphased		
POG1068	*TERT*	chr5	1295113	G	A	HP2		

Table S6. TERT mutations in long-read POG cohort and associated expression and allele-specific methylation, related to Figure 5.

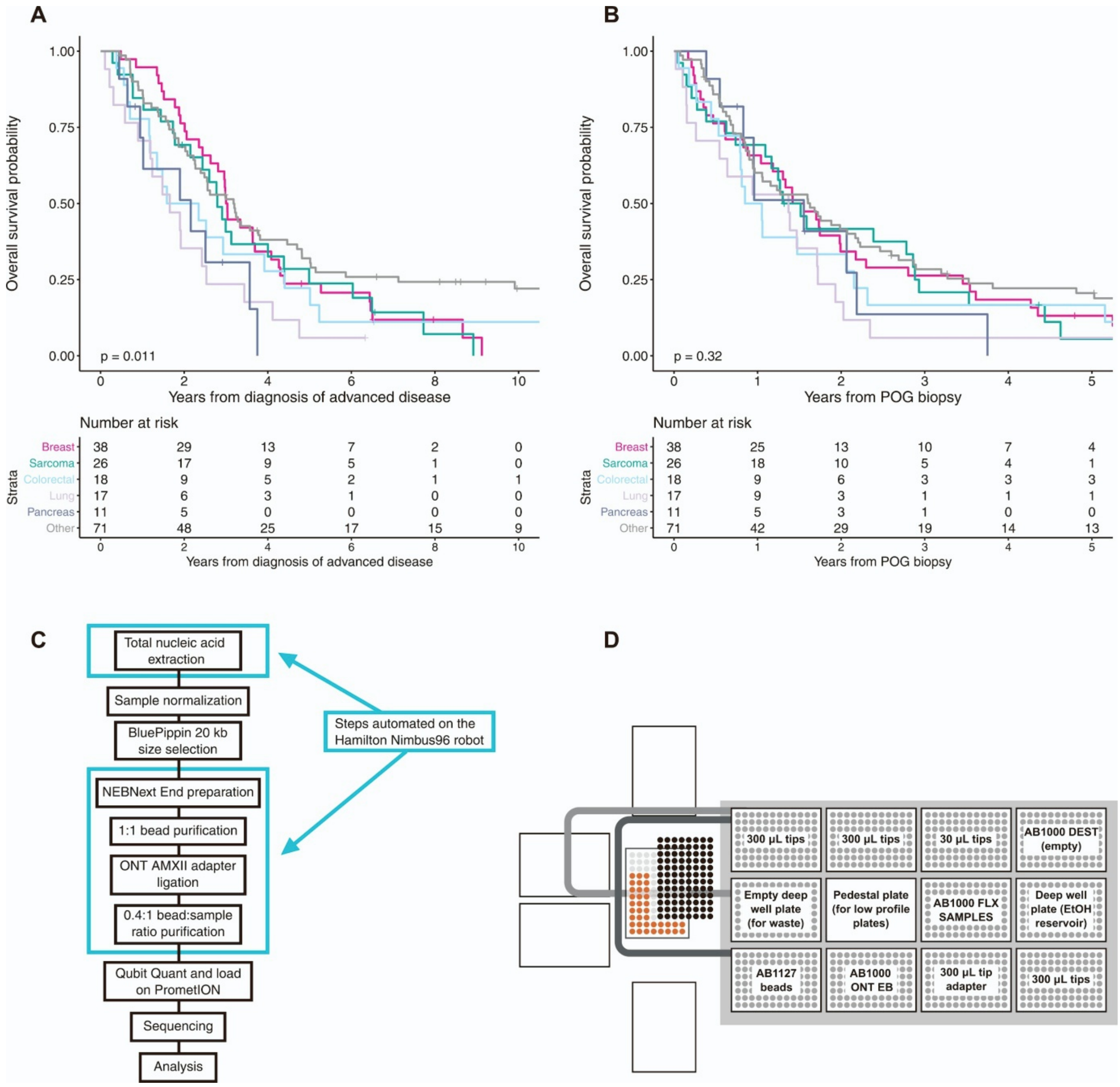

Figure S1. Cohort and sequencing information, related to Figure 1. (A-B) Overall survival for Long-read POG cohort by tumour type from date of advanced disease (A) and from date of biopsy (B). (C) Workflow for Nanopore sample preparation highlighting steps automated on the Nimbus96 liquid handler. (D) Nimbus96 robot deck layout for magnetic bead purification.

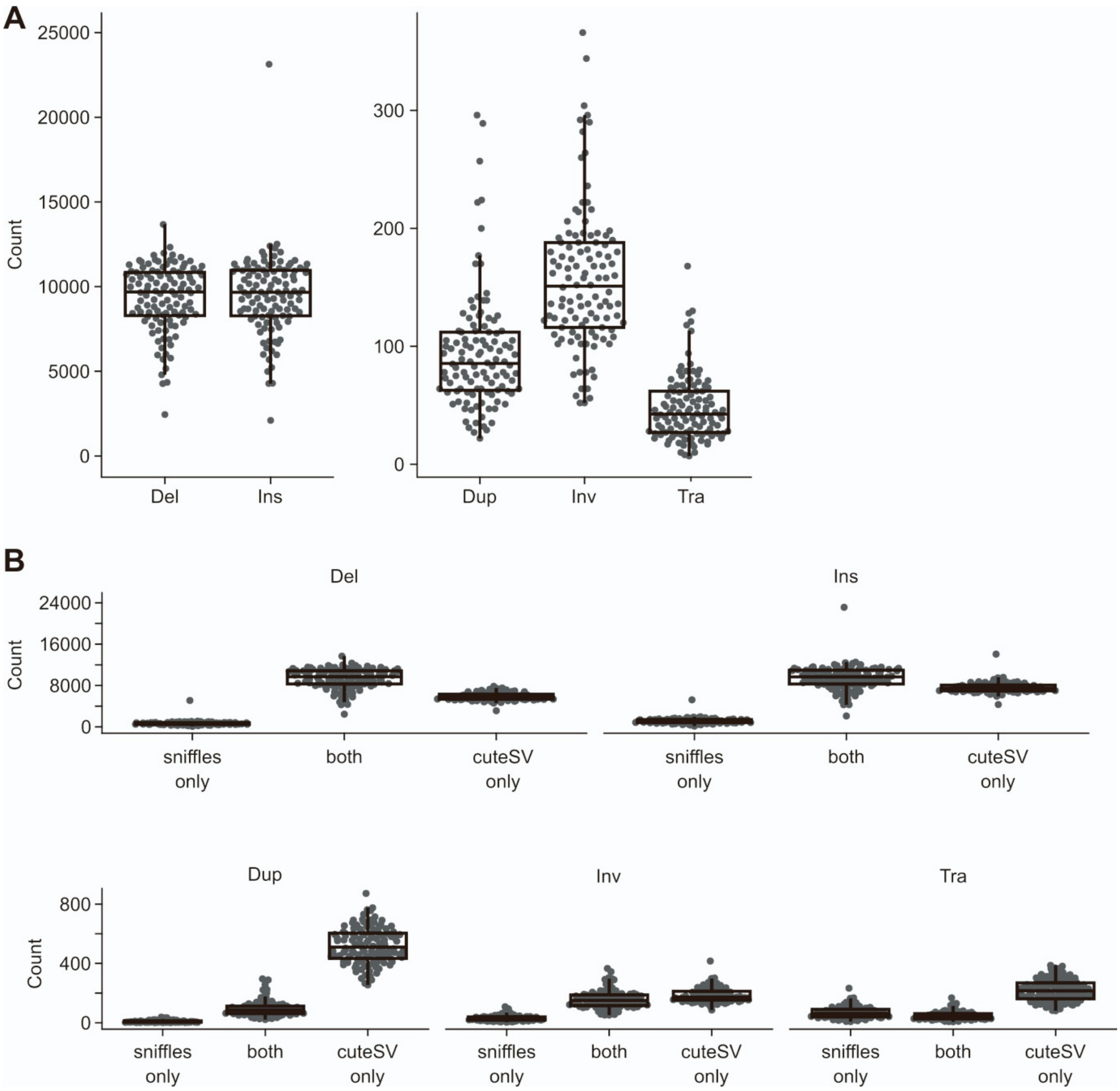

Figure S2. Per-sample counts of all SV calls (including germline) by type for all tumours, related to Figure 2. (A) Consensus calls between cuteSV and sniffles. (B) SV calls further broken down by caller across the cohort (cuteSV vs sniffles, vs calls shared between both callers).

A

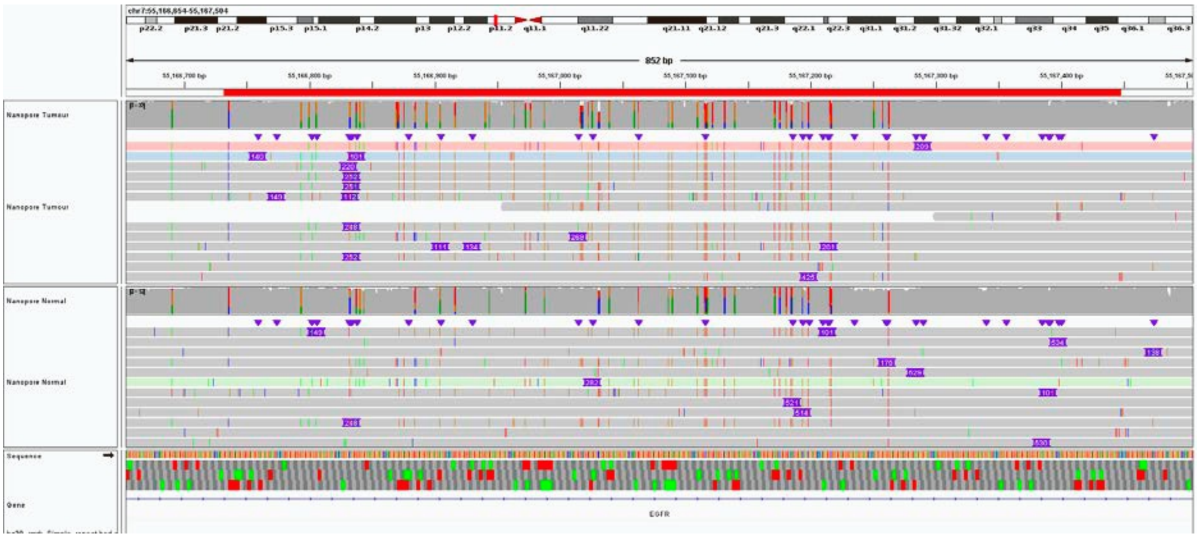

B

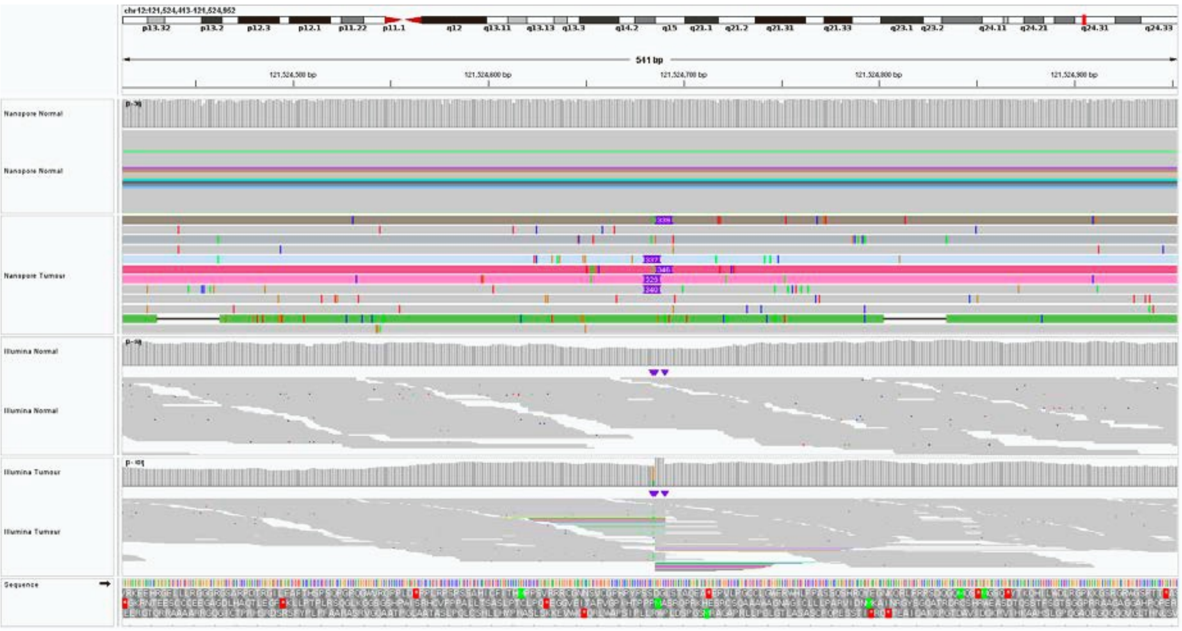

Figure S3. Insertion events called in long-read data but not in short-read, related to Figure 2. (a) Putative false positive intronic 90 bp insertion event on *EGFR* at chr7:55167011 in POG049 called by nanomonSV with no visible read based support for a 90 bp event. (b) Intronic 328 bp insertion event on *KDM2B* at chr12:121524684 called by nanomonSV in POG111, miscalled by Illumina as a 111,113 bp inversion around chr12:121413571-121524684.

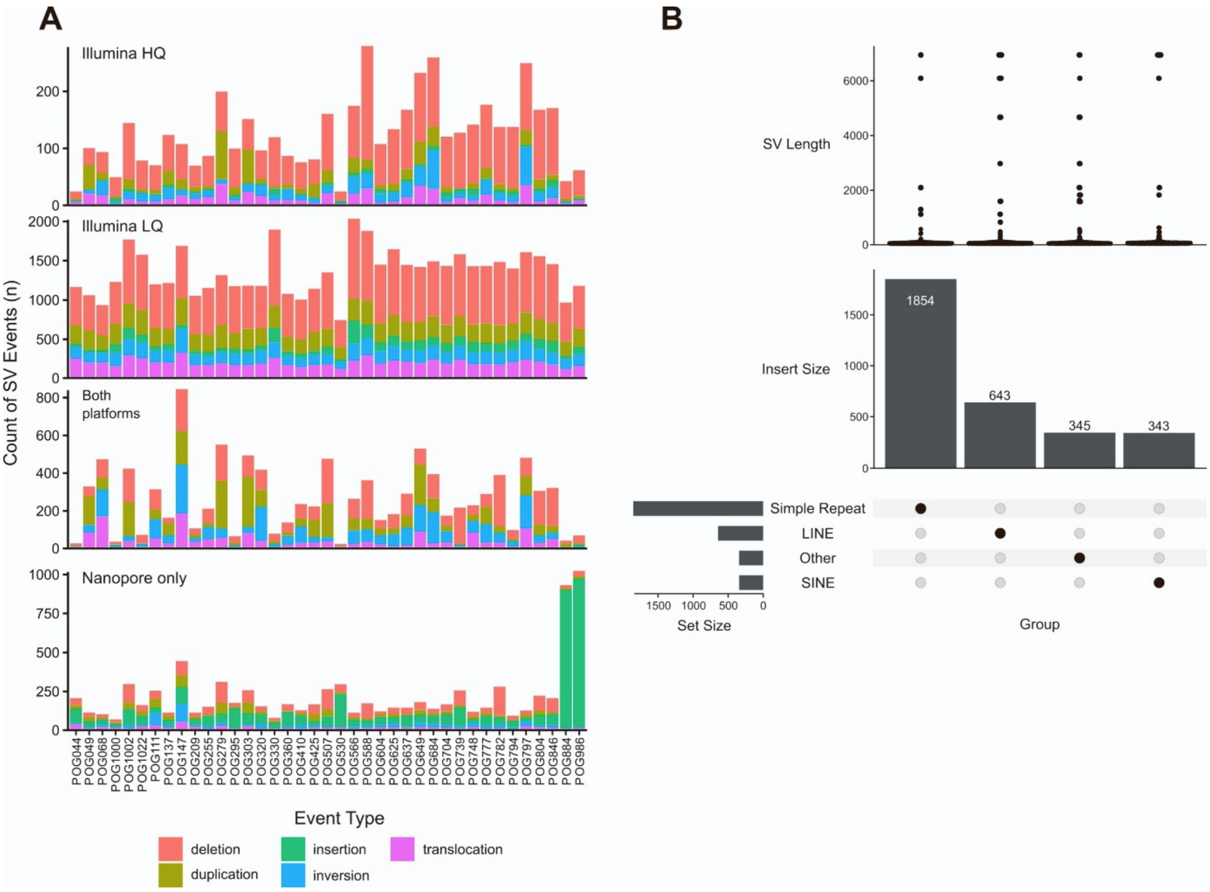

Figure S4. SV distributions and examples, related to Figure 2. (A) Number of somatic SV events per sample in different call sets. POG111 and POG147 stand out as having much more inversions called in the long-read data than all other cases. POG884 and POG986 stand out as having much more insertions called. (B) Nanopore-only insertion calls in an MSI-H sample POG986, annotated with RepeatMasker. Other repetitive elements such as Alu elements within RepeatMasker are demarcated as “other”

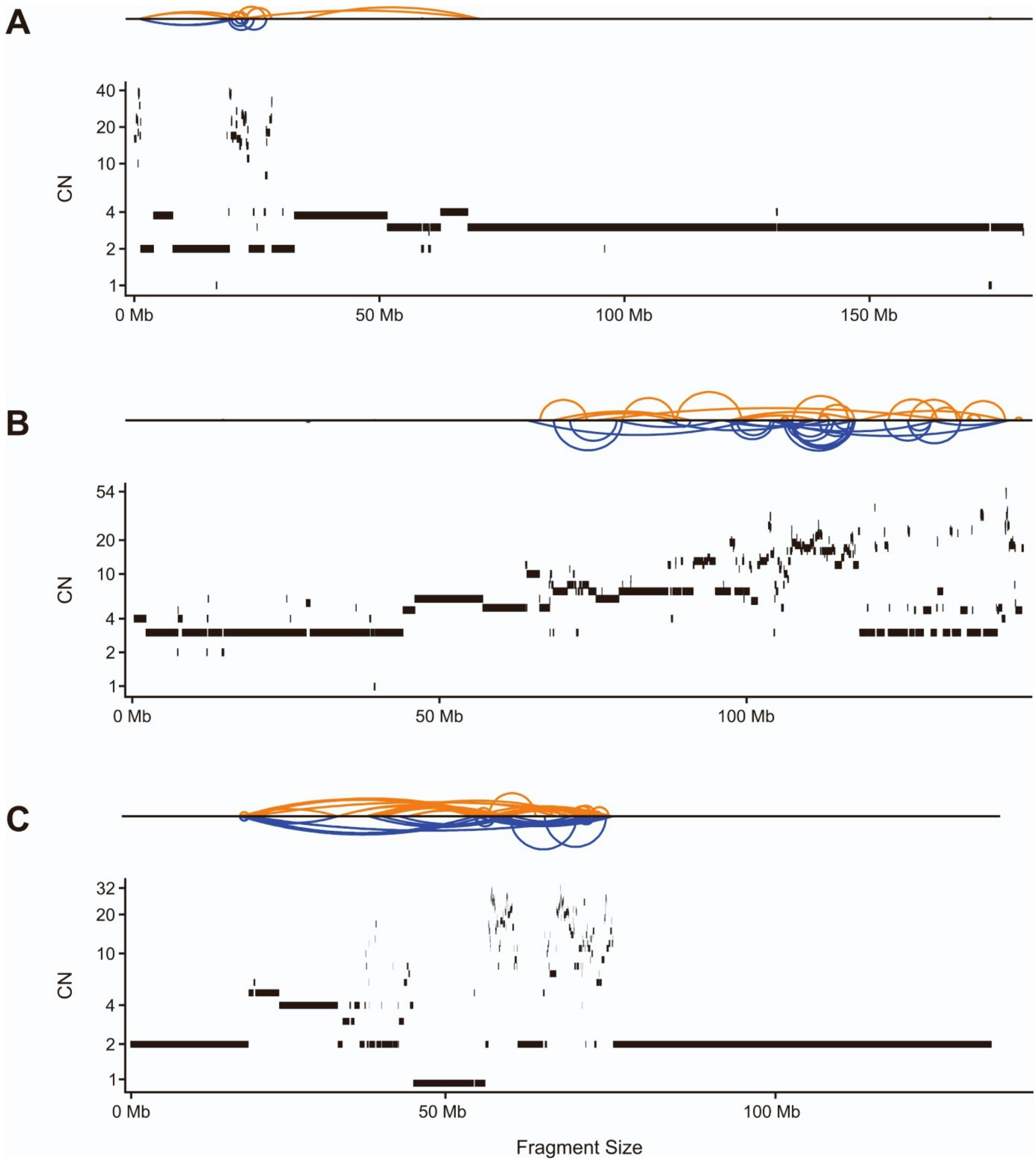

Figure S5. Examples of complex SVs, related to Figure 2. (A-C) Shatterseek images of patient POG147 (A) and patient POG111 (B,C) with the 'Sniffles' structural variant profiles (top of subplots), and Ploidetect copy number calls (bottom of subplots). Duplication-like SVs are coloured blue, and deletion-like SVs are coloured orange, and highlight the tyfona-like structural variant profile of chromosome 5 (A) and chromosome 8 (B,C).

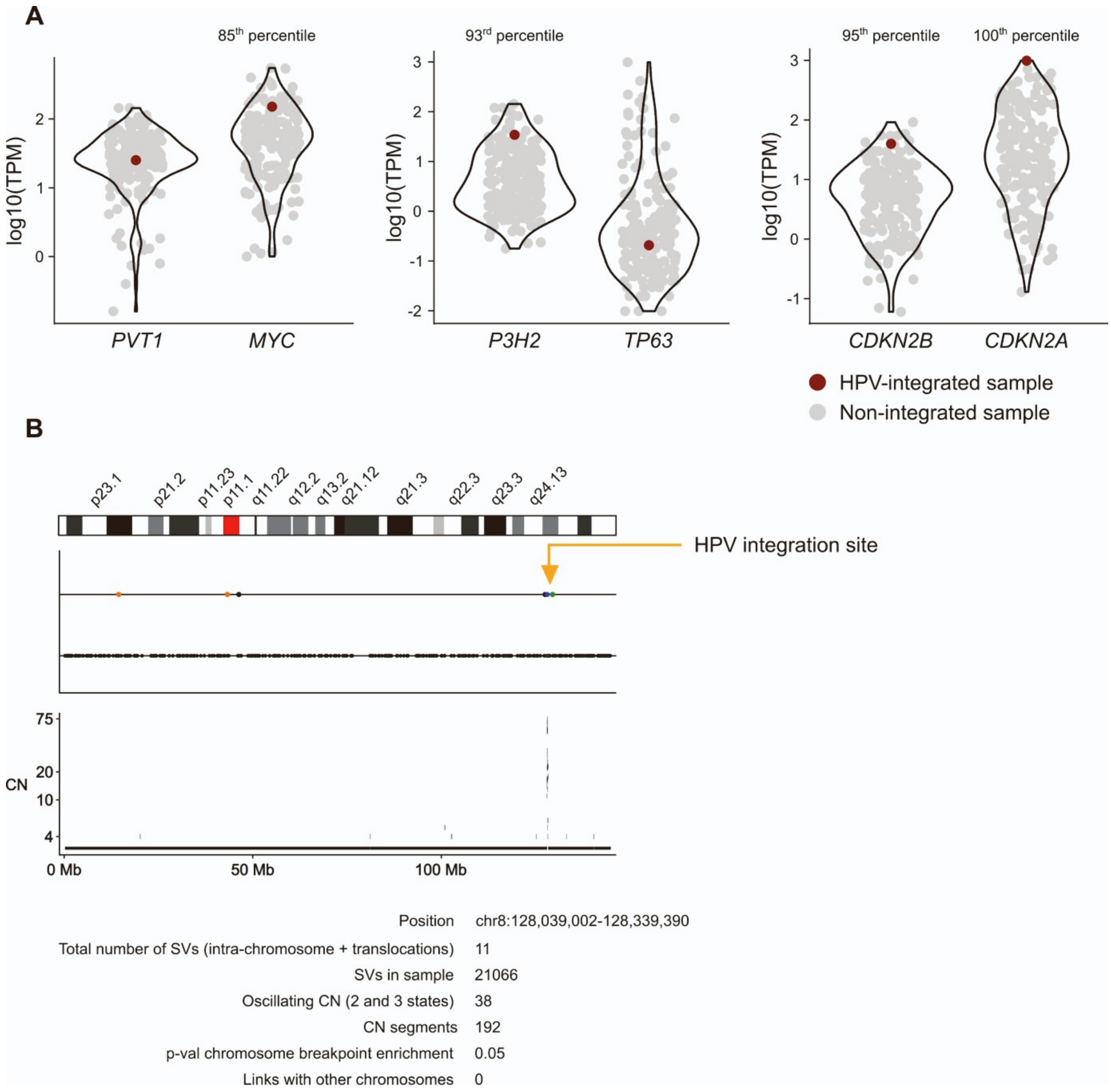

Figure S6. HPV integration events, related to Figure 2. (a) Gene expression in HPV-integrated samples. (b) Focal chromothripsis-like event at HPV integration site.

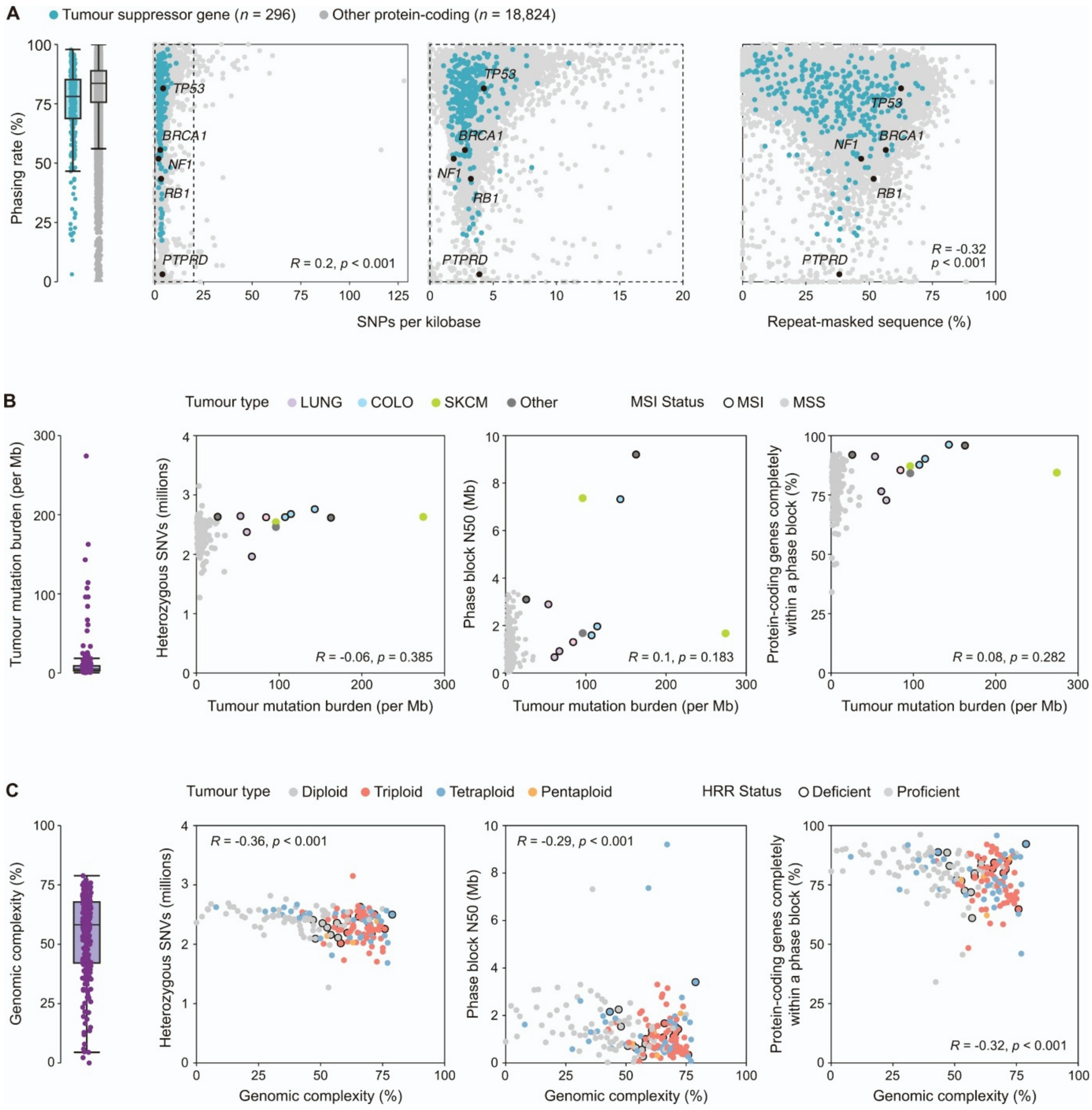

Figure S7. Factors affecting phasing in long-read sequenced tumours, related to Figure 3. (A) Effect of germline factors on phasing rate per-gene. SNP density (left) and proportion of the gene body made up of repeats (right) both correlate weakly with phasing rate. (B) Effects of somatic small variants (represented by TMB) on phasing. Neither phase block N50 nor completeness of phasing were significantly correlated. (C) Effect of somatic rearrangements (measured by genomic complexity) on phasing. Both phase block N50 and completeness of phasing were correlated, slightly more so than SNP density and repeats.

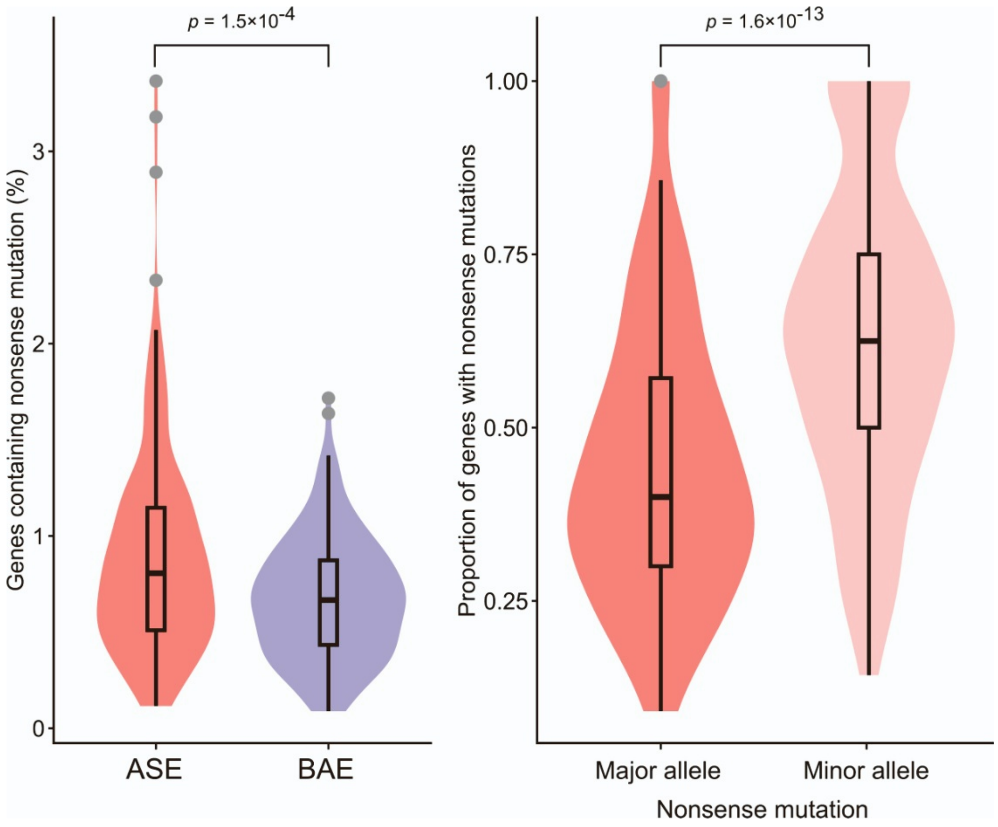

Figure S8. ASE in genes with nonsense mutations, related to Figure 3. (A) ASE status of genes containing nonsense mutations. (B) ASE genes with nonsense mutations by major and minor expressed allele. P values are Wilcoxon rank sum test.

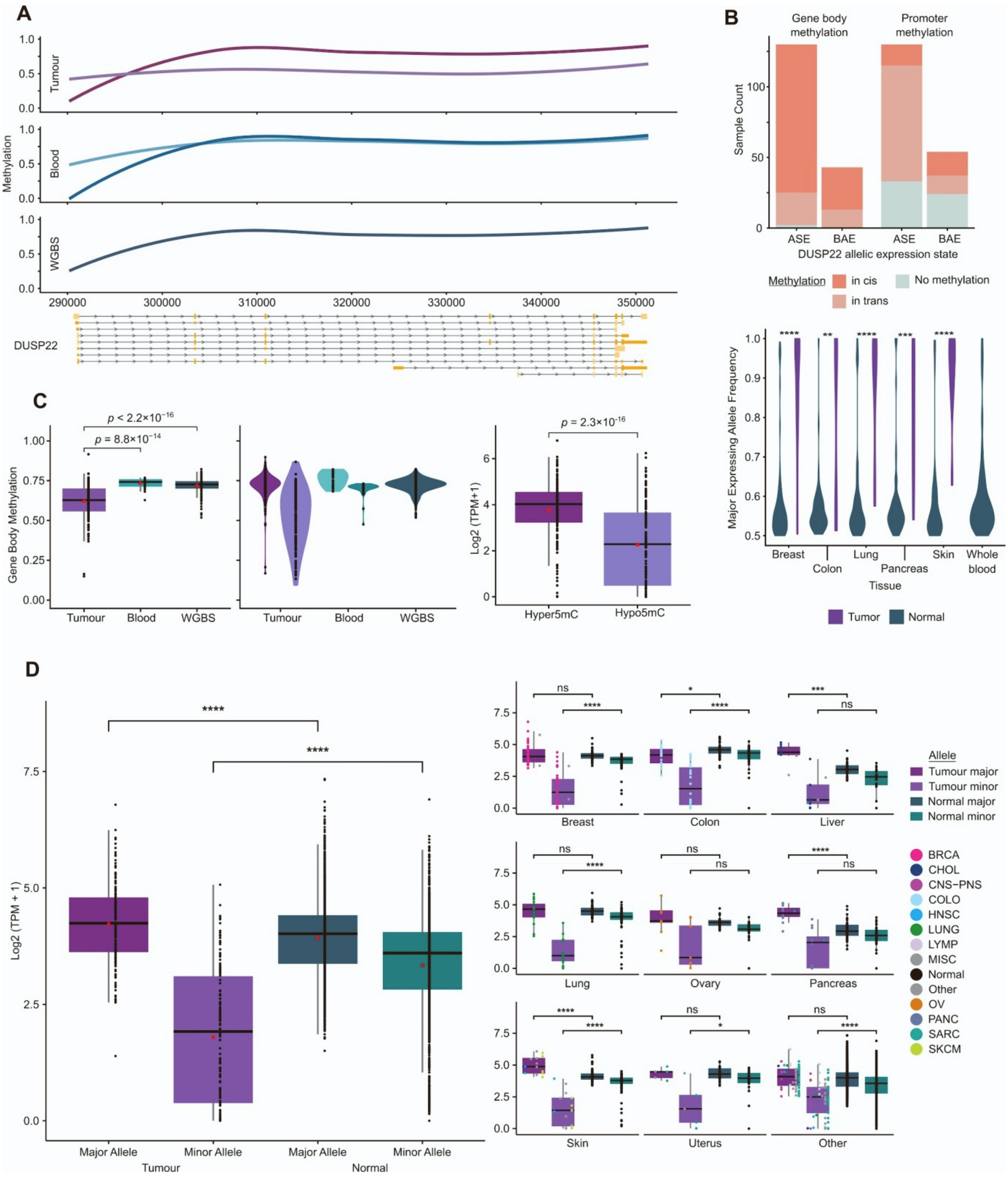

Figure S9. Phasing of methylation information, related to Figure 4. (A) Allelic methylation of CpGs across *DUSP22*. Lighter points indicate hypomethylated alleles across the region and darker indicate hypermethylated alleles. Bottom, gene tracks for the top ten expressed transcripts. (C) *DUSP22* gene body methylation of tumour, blood and normal whole genome bisulfate sequencing samples at the sample and allelic level respectively (left two figures). Expression of alleles showing gene body hypermethylation in comparison to those showing hypomethylation (right). (D) Allelic expression of *DUSP22* for the major and minor allele of tumor samples compared to GTEx samples with more than 20 reads to determine major allele frequency. Allelic expression is shown across tissues of origin for cancers and normals and the color of each point indicates the cancer type. Wilcoxon rank-sum test p-values and significance labels are shown above plots (P<0.0001: ****, P<0.001: ***, P>0.05: ns). All samples with loss of heterozygosity or deletions in this gene were filtered out.

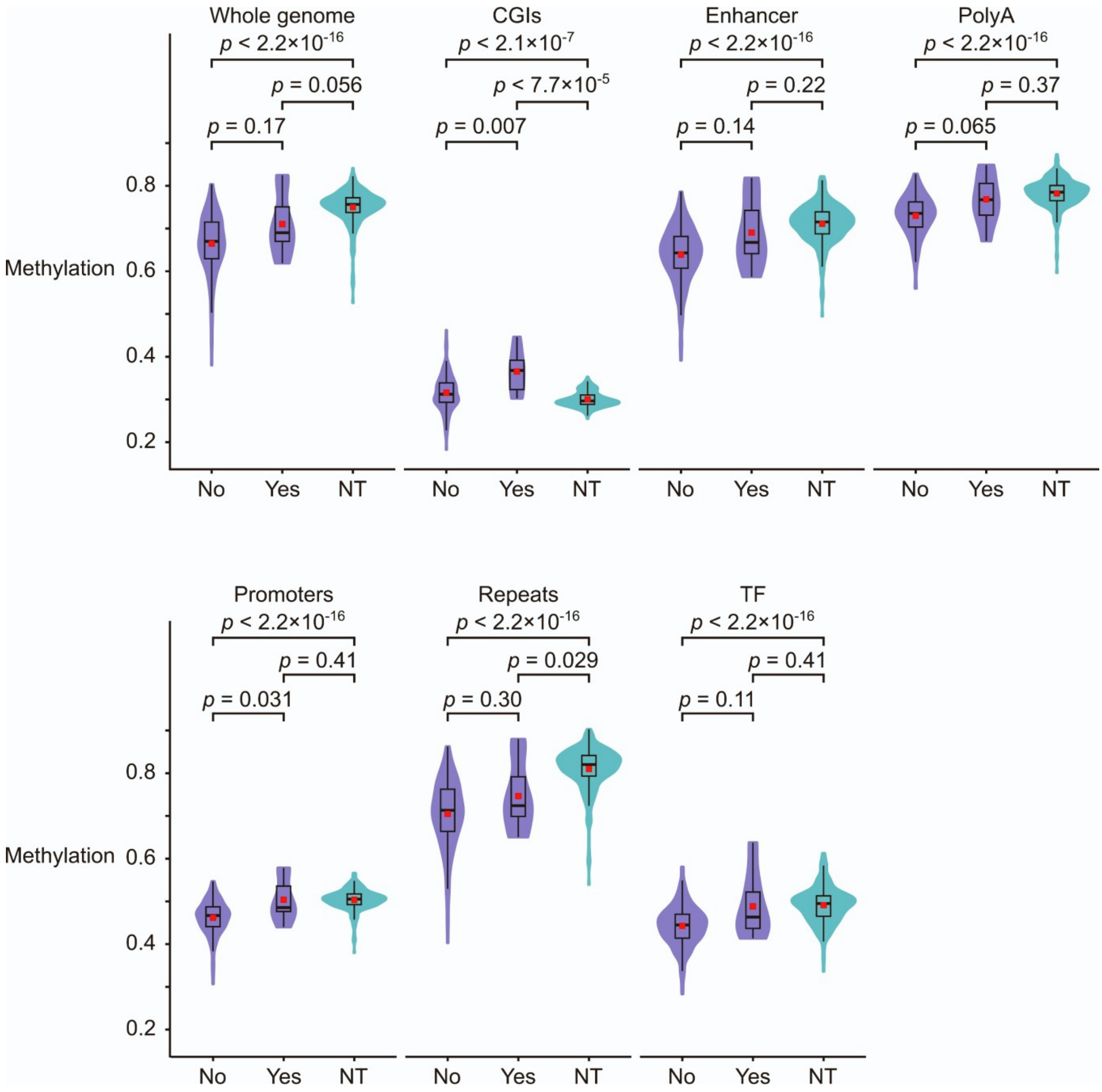

Figure S10. Average methylation at a range of regulatory regions in POG cases with either IDH activating or TET-inactivating mutations, related to Figure 4.

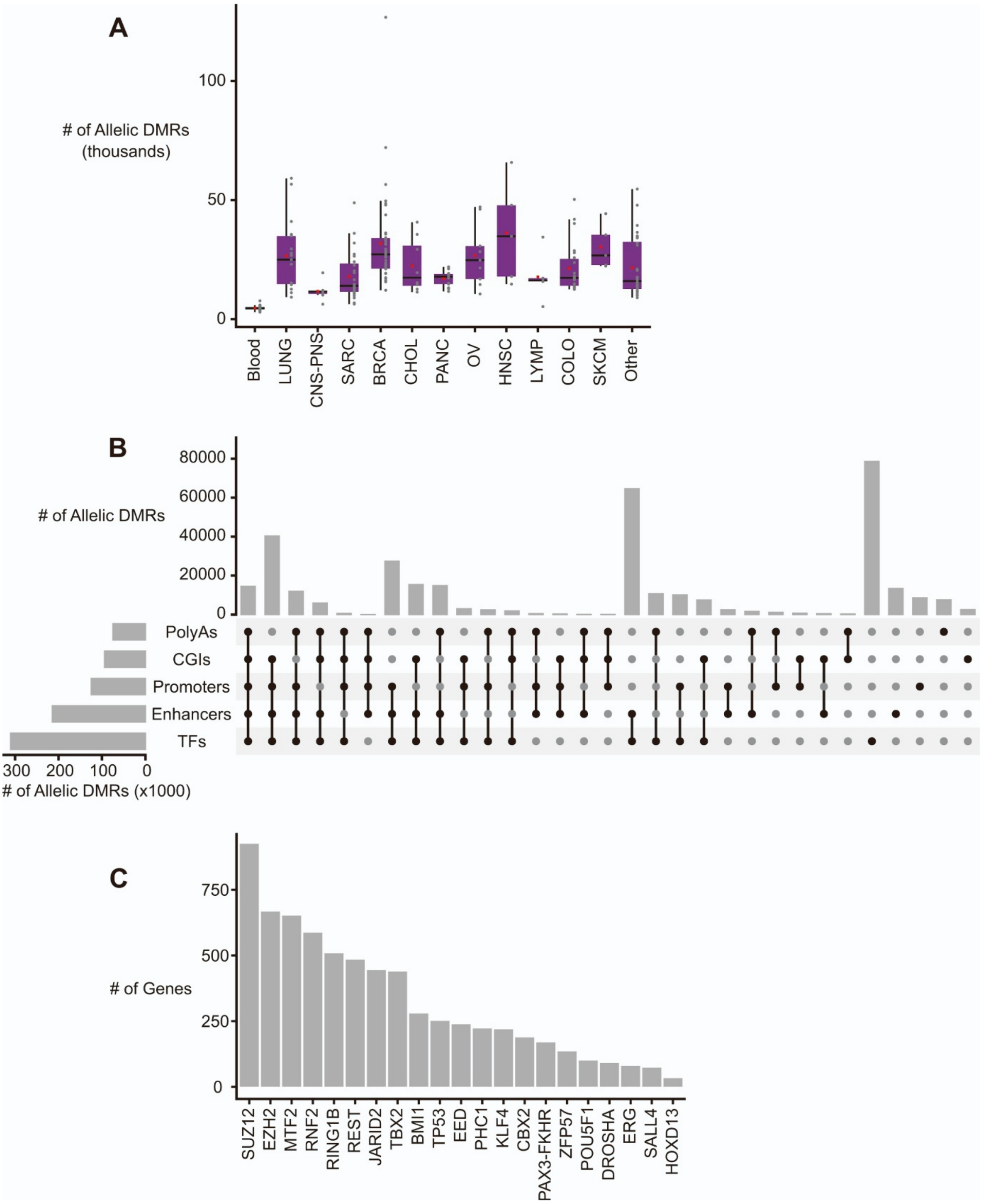

Figure S11. Allele-specific DMRs, related to Figure 4. (a) Number of allele-specific DMRs in matched blood and tumour type samples. (b) Upset plot demonstrating mapping of tumour-specific allelic DMRs to different genomic regions. (c) Recurring transcription factor (TF) binding sites with tumour-specific allelic DMR. The plot represents the top 30 TFs with the highest proportion of their binding sites recurrently overlapped to tumour-specific allelic DMR.

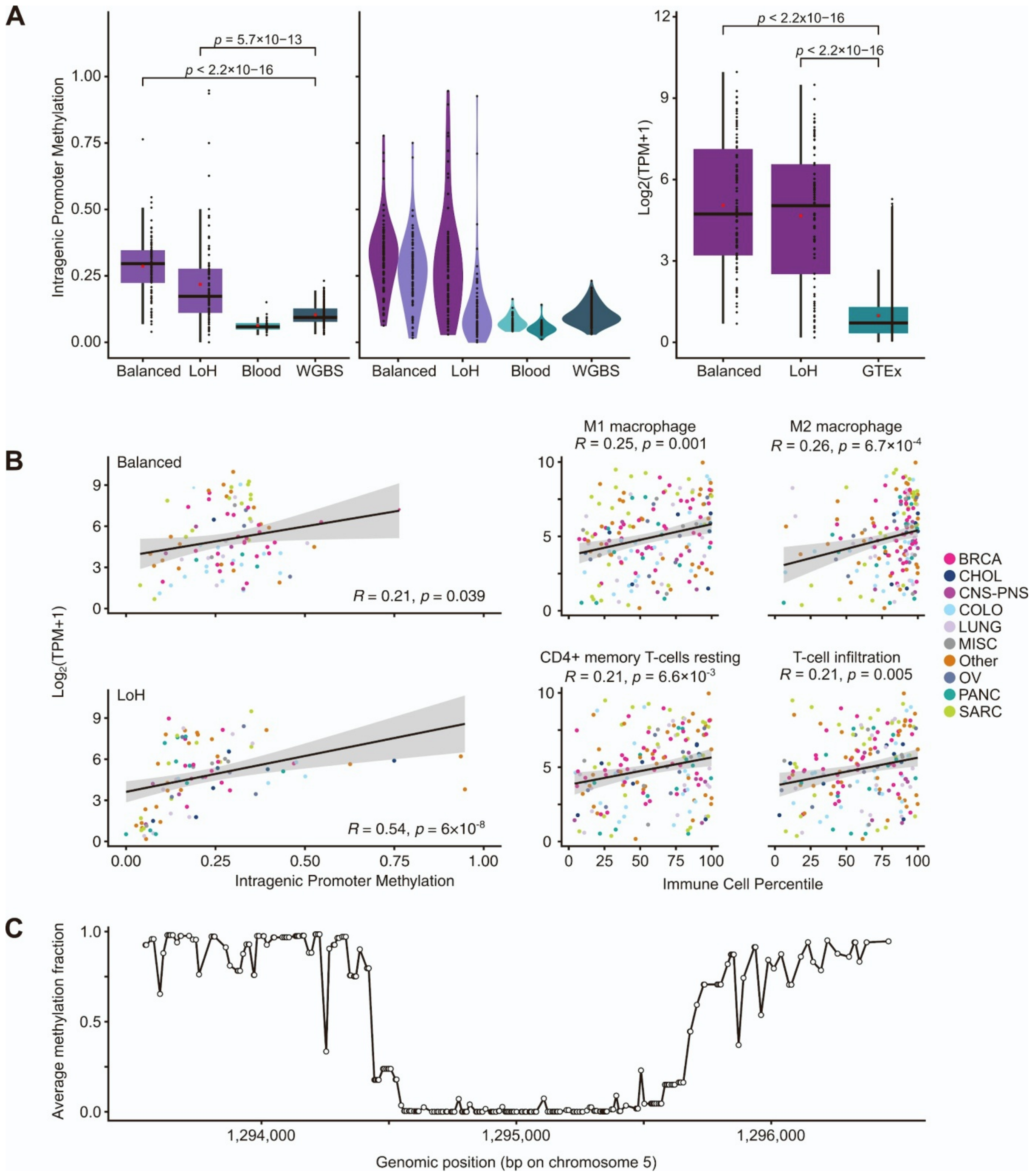

Figure S12. Methylation of specific cancer genes, related to Figure 5. (A) *CDKN2A* intragenic promoter methylation of balanced tumour, loss of heterozygosity (LOH) tumour, blood and normal reference samples at the sample (left) and allelic (centre) level. Expression of balanced and LOH tumor samples in comparison to Genotype-Tissue Expression (GTEx) reference expression data for all tissues (right). *P*-values are Wilcoxon rank-sum test. WGBS, whole genome bisulfite sequencing. (B) *CDKN2A* intragenic promoter methylation and expression for balanced and LOH samples (left). Percentile score for immune cell populations inferred by CIBERSORT in comparison to *CDKN2A* expression per sample (right). Spearman R values and corresponding p-values are shown. (C) Average fraction of methylated bases at each CpG in TERT promoter region in POG blood normals.

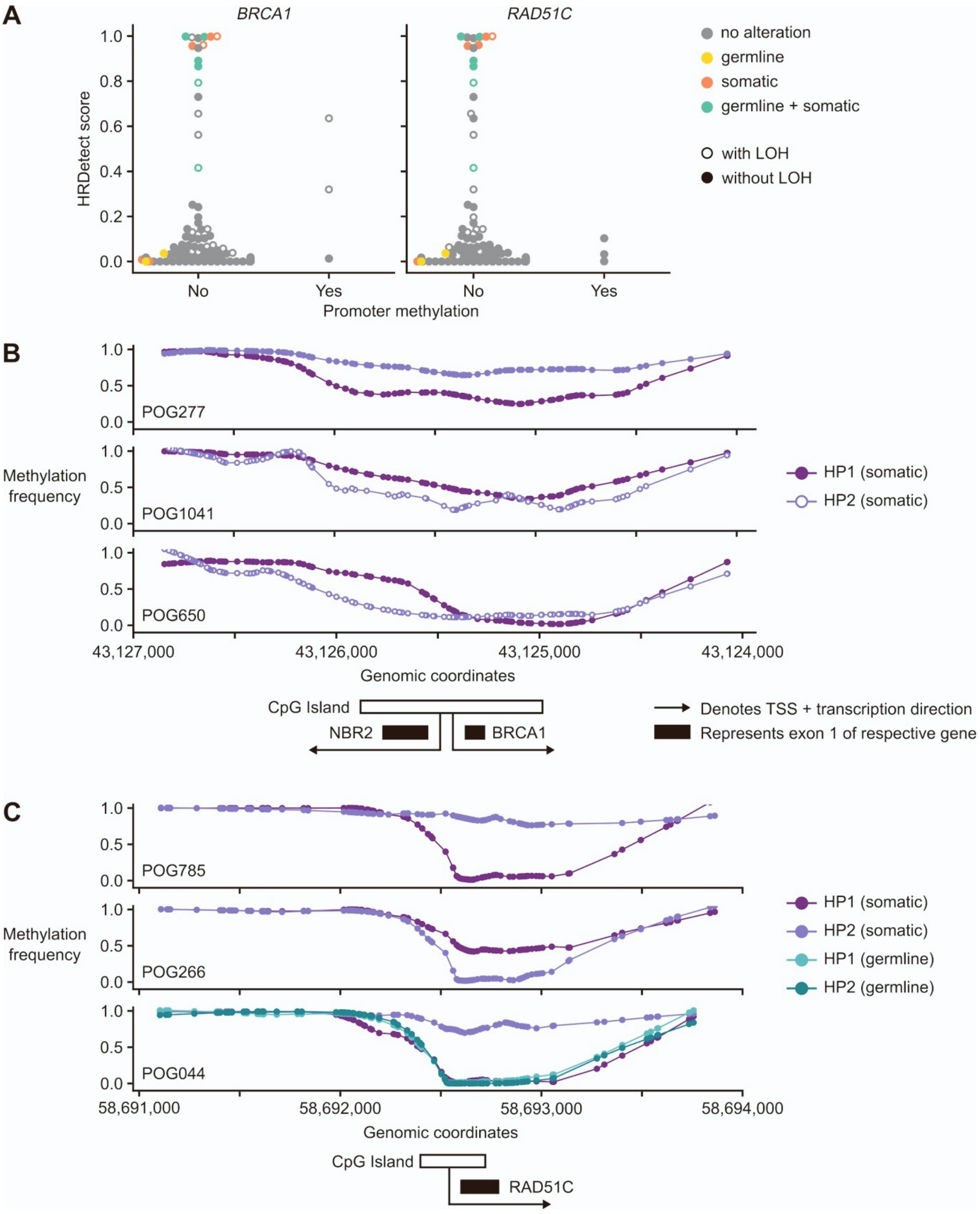

Figure S13. Methylation at tumour suppressor promoters, related to Figure 6. (a) Somatic mutation status and promoter methylation in *BRCA1* and *RAD51C*. (b) Allele-specific methylation in the *BRCA1* promoter region in three cases, POG277, POG1041, POG650. (c) Allele-specific methylation in the *RAD51C* promoter region in three cases, POG785, POG266, POG044.

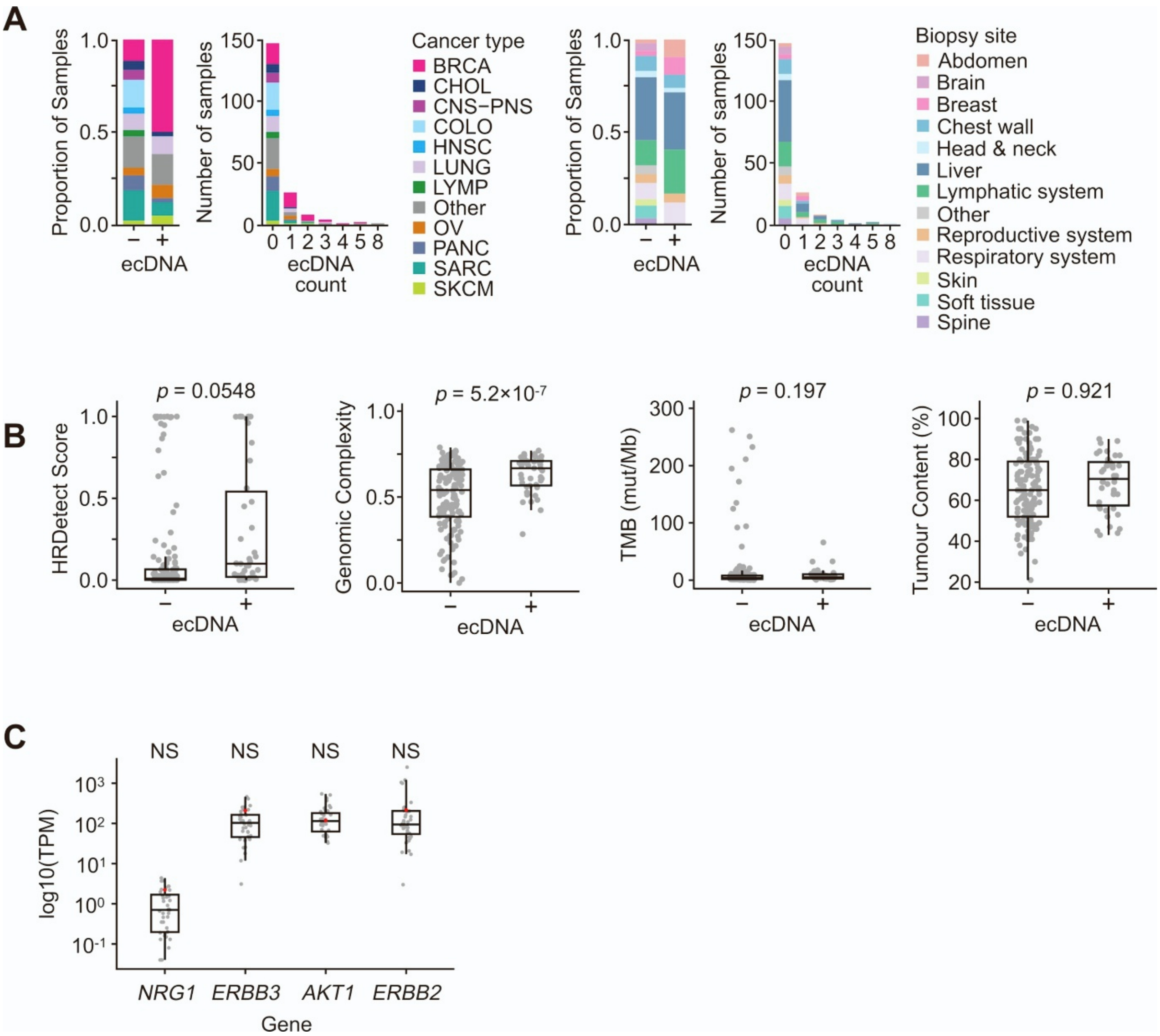

Figure S14. Summary of ecDNAs detected across cohort, related to Figure 6. (A) Proportion of ecDNA+ and ecDNA- samples stratified by cancer type and biopsy site. Results obtained from running 189 samples through AmpliconArchitect, a short-read WGS ecDNA detection tool. (B) Molecular correlates of ecDNAs (n = 189). Two-sided Student's *t*-tests were used to judge significance with Bonferroni multiple testing correction. (C) Expression in transcripts per million (TPM) for *NRG1* pathway genes for ecDNA-containing breast cancer sample shown in red (*n*=1) compared to other breast cancer samples in the cohort (*n*=39). Significance assessed via one-vs-all permutation tests, with Bonferroni multiple testing correction.
